# Global Research Trends and Insights on AI Usage in Tuberculosis: Bibliometric Analysis

**DOI:** 10.2196/93885

**Published:** 2026-09-10

**Authors:** Jiawen He, Shuzhen Feng, Yufang Chen, Zhijuan Lai, Beihua Lyu, Yingying Li

**Affiliations:** 1 Guangdong Pharmaceutical University, No. 283, Jianghai Avenue, Haizhu District, Guangzhou, Guangdong, 510006, China, 86 1-562-277-9423

**Keywords:** artificial intelligence, AI, bibliometric analysis, deep learning, medical imaging analysis, tuberculosis

## Abstract

**Background:**

Tuberculosis (TB) remains a major global health challenge despite prevention efforts. AI offers promising approaches to long-standing challenges in TB diagnosis, drug resistance detection, and case management; however, a systematic mapping of global research priorities and translational gaps in AI applications to TB is currently lacking.

**Objective:**

This study aimed to analyze development patterns, collaboration networks, and knowledge structure of AI applications in TB management through bibliometric analysis of Web of Science publications, identifying research frontiers and translational gaps.

**Methods:**

This study conducted a bibliometric analysis of Web of Science Core Collection publications (2000‐2025; last searched April 4, 2025). Original articles and reviews containing both AI- and TB-related terms were screened by 2 independent reviewers following PRISMA 2020 principles. Coauthorship, cocitation, and keyword cooccurrence networks were constructed with VOSviewer (Nees Jan van Eck and Ludo Waltman, Leiden University), CiteSpace (Dr. Chaomei Chen, Drexel University), and Bibliometrix R package (Massimo Aria and Corrado Cuccurullo, University of Naples Federico II), and results were synthesized through descriptive statistics and scientific knowledge mapping.

**Results:**

Analysis of 1300 articles shows substantial growth in AI applications for TB management during 2000‐2025, with post-2018 publication growth of 29.7% and citation growth of 54.2% annually. The United States dominated output (346 publications; 12,143 citations), whereas high-burden countries remained underrepresented relative to disease burden, revealing a systematic research-demand inversion. Of the 15 most cited papers, 4 (26.7%) focused on imaging analysis, 2 (13.3%) on drug resistance prediction, and 3 (20.0%) on drug discovery, together comprising 60% (9/15) of the highly cited literature; the remainder addressed foundational biology, decentralized-learning methodology, immunoinformatics, and review literature rather than a specific TB application domain. Across this literature, technical validation–stage work markedly outweighed papers reporting clinical application, underscoring a technology-over-translation imbalance; key gaps persist in cross-regional data sharing, atypical lesion recognition, and socioeconomic factor integration. These findings indicate that clinicians and program managers in high-burden settings should prioritize locally validated, implementation-ready AI tools over algorithmic refinements, and that funders should direct resources toward prospective clinical trials and equity-focused deployment frameworks.

**Conclusions:**

AI-TB research has expanded rapidly, yet clinical translation remains disproportionately limited relative to algorithmic innovation. To help bridge this gap, frontline clinicians in high-burden settings could prioritize locally validated computer-aided detection (CAD) tools meeting World Health Organization (WHO)–recommended performance standards for chest-radiograph triage, and contribute real-world performance data through postdeployment surveillance channels; policymakers could support cross-national data-sharing frameworks and consider integrating validated AI tools into national TB screening programs; and funders could allocate a defined share of AI-for-TB grants to prospective clinical evaluation rather than further algorithmic refinement alone. These findings should be interpreted in light of the study’s reliance on a single English-language database and the inherent limitations of bibliometric proxies, which may underrepresent research from high-burden regions and undervalue recent publications.

## Introduction

Tuberculosis (TB) remains a leading cause of infectious-disease mortality worldwide. According to the 2024 World Health Organization (WHO) Global Tuberculosis Report, following the recession of the COVID-19 pandemic, TB has probably regained its position as the leading cause of death from a single infectious agent, with more than one million deaths reported in 2023; the global treatment success rate for drug-resistant TB has persisted below 70% [1,2]. Conventional case detection, drug-resistance diagnosis, and case management are constrained by shortages of trained radiologists, the multiweek turnaround of phenotypic drug-susceptibility testing, and limited adherence support, particularly in high-burden, resource-limited settings.

These constraints have motivated an active line of AI research in TB over the past decade, organized around 3 application domains. Computer-aided detection (CAD) of TB on chest radiographs has progressed from research prototypes toward programmatic implementation and was recommended in the 2021 WHO consolidated guidelines as an alternative to human reading for screening individuals aged 15 years or older [3]. Machine learning models trained on *Mycobacterium tuberculosis *whole-genome sequencing data have been developed to predict resistance to first- and second-line drugs [4]. Deep learning approaches to antibacterial discovery have identified compounds with in vitro activity against *M. tuberculosis* and other multidrug-resistant pathogens [5], a development of particular relevance given the long-standing scarcity of novel anti-TB drug classes. Despite this growth, the field exhibits internal imbalances, uneven geographic distribution of research output relative to disease burden, concentration of effort in algorithmic refinement over clinical deployment, and limited integration of socioeconomic determinants, whose scope and structure have not been systematically characterized.

Although bibliometric analyses have separately examined TB research trends [6,7] and AI applications in health care [8,9], the AI-TB intersection has, to our knowledge, not been systematically characterized. This study addresses this gap by applying 3 complementary tools—VOSviewer (Leiden University), CiteSpace (Dr. Chaomei Chen, Drexel University), and Bibliometrix R (Posit PBC)—to the full 2000‐2025 Web of Science Core Collection (WOSCC) corpus of AI-TB publications, in order to (1) characterize the development trajectory and collaboration topology of the field, (2) examine the geographic relationship between research output and TB disease burden, and (3) identify concrete translational gaps. The resulting evidence base is intended to inform research priority-setting and policy discussions in global TB control.

## Methods

### Literature Sources and Search Strategy

The data for this study were sourced from the WOSCC [10]. As a globally recognized authoritative academic citation database, WOSCC has become the gold standard in bibliometric research due to its rigorous journal selection criteria (covering 254 disciplines across natural sciences, social sciences, and arts and humanities) and high-quality content inclusion (with more than 21,000 scholarly journals that undergo strict peer review). Numerous empirical studies have confirmed the effectiveness and reliability of its data in citation network analysis [11] and the prediction of disciplinary development trends [12], especially its citation indexing mechanism, which provides core data support for the construction of scientific knowledge maps. The search query used for this study is as follows: Topic Search (TS)=(“Artificial Intelligence” OR"Machine Intelligence” OR “Machine Learning” OR “Supervised Machine Learning” OR “Unsupervised Machine Learning” OR “Deep Learning” OR “Neural Networks*” OR “Decision Trees*” OR"Computer Vision System"OR “Intelligent System*” OR “Support Vector Machines*” OR “Predictive Modeling” OR “Feature Extraction” OR “Data Mining” OR “Generative Artificial Intelligence"OR “Large Language Model*” OR “Transformer Model*“) AND TS=(“Tuberculosis” OR “Latent Tuberculosis” OR “Multidrug-Resistant Tuberculosis” OR “Extensively Drug-Resistant Tuberculosis” OR “Pulmonary Tuberculosis” OR “Extrapulmonary Tuberculosis” OR “Central Nervous System Tuberculosis” OR “Meningeal Tuberculosis” OR “Spinal Tuberculosis” OR “Miliary Tuberculosis” OR “Mycobacterium tuberculosis”). The retrieval period was January 1, 2000, to April 4, 2025; this cutoff ensured complete indexing and citation processing for all included records, as publications after this date would carry incomplete metadata and under-developed citation counts. Restricting the search to WOSCC may underrepresent research from high-burden regions publishing in regional or non-English databases; our findings therefore characterize internationally visible, English-language AI-TB research.

1. Eligibility criteria: Inclusion criteria were—WOSCC-indexed original articles or reviews in English containing at least one AI- and one TB-related term from the search query. Records were excluded if they were editorials, letters, conference papers, meeting abstracts, news items, retractions, early-access records, or book chapters; if AI- or TB-related terms appeared only incidentally; if they were duplicates from multiedition indexing; or if metadata were incomplete.
2. Screening process: Two reviewers (JH and SF) independently assessed eligibility following the PRISMA (Preferred Reporting Items for Systematic Reviews and Meta-Analyses) 2020 transparency principles [13], with discrepancies resolved by a third reviewer (YL); off-topic records were filtered during data cleaning. As this is a bibliometric study, individual-article quality appraisal was not performed; research quality was instead operationalized through citation count, journal impact factor (IF), and h-index. Highly cited papers were categorized into 3 application domains—diagnostics, therapeutics, and public health—based on each paper’s primary objective.

### Research Tools and Methods

#### Overview

After data cleaning, the dataset was standardized and exported in Microsoft Excel and TXT formats, retaining key metadata fields (title, authors, keywords, affiliations, country or region, cited references, source journal, and publication date). A total of 3 complementary bibliometric tools were applied in parallel. VOSviewer (version 1.6.18) [14], was used to construct country or region, institutional, author, and keyword cooccurrence networks, applying full counting normalization with minimum thresholds of 5 occurrences for keywords, 4 publications for authors, 7 for institutions, and 10 for countries or regions. CiteSpace (versions 6.3.R3 through 6.4.R2; applied at successive analytical stages) [15,16] for detecting emerging trends and citation bursts in scientific literature was used for journal dual-map overlay, document cocitation analysis, burst detection, and keyword burst analysis, with log-likelihood ratio (LLR) cluster labeling and a one-year time slice. The Bibliometrix R package (versions 3.2.1 and 4.0.2; Posit PBC) [17-19] was used for descriptive statistics and country-level scientific production analysis.

#### Keyword Processing

**Figure 1.**
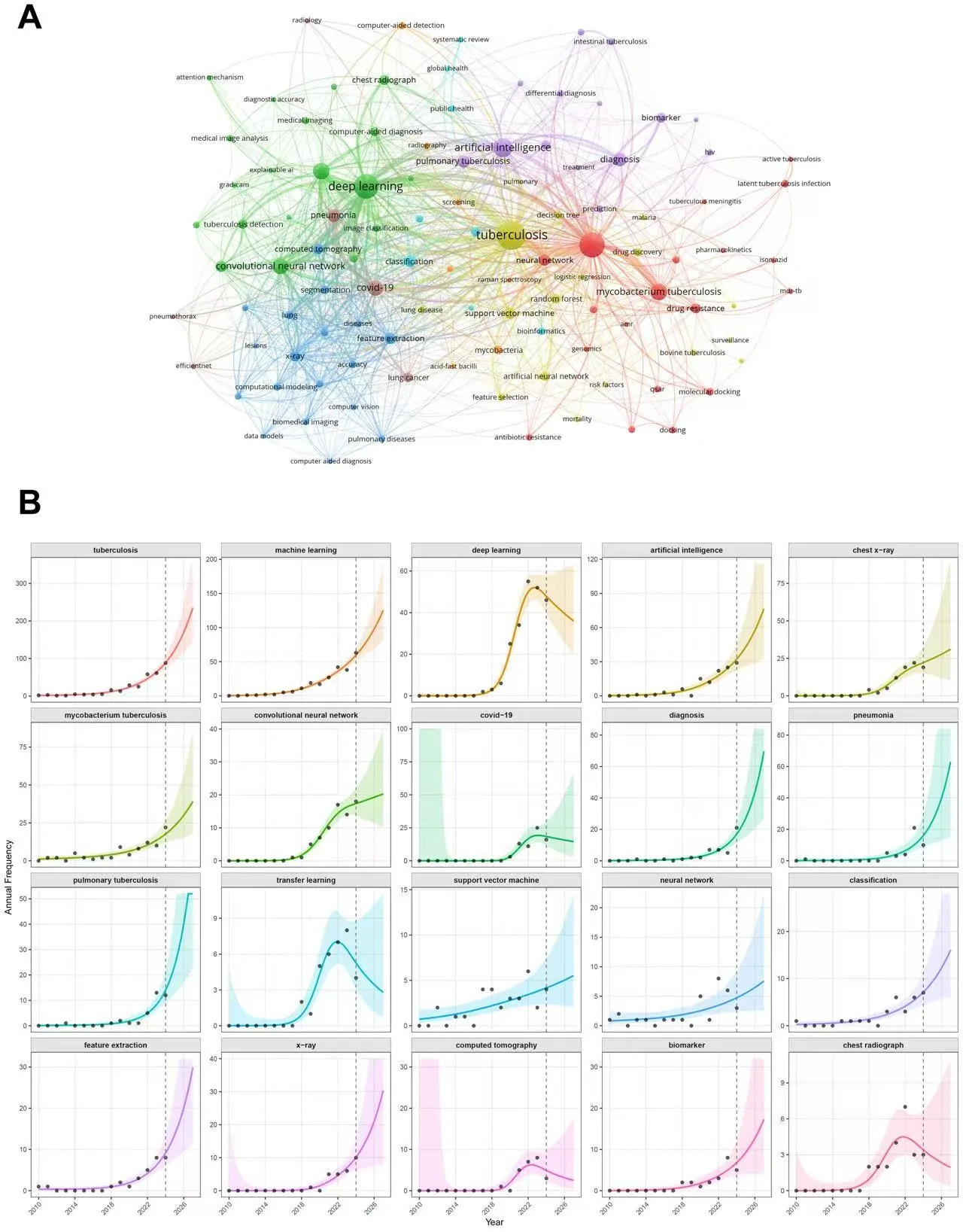
Keyword cooccurrence network, temporal trends, and future forecasts of the top 20 keywords in AI-tuberculosis (TB) research (2010‐2027). (A) Network visualization of the most frequently occurring keywords and their relationships. Different research directions are distinguished by color, and the size of the nodes reflects the frequency of occurrence. (B) Temporal trends and future forecasts of the top 20 keywords (2010‐2027). Each sub-panel displays the annual frequency trajectory for a specific high-frequency keyword within the AI and TB domain. Circular markers represent the observed article-level annual frequency for each given year. Solid colored curves indicate the fitted historical trend and forward projection calculated via robust generalized additive models (GAM). Shaded ribbons represent the capped 95% CIs for the trend estimates. The vertical dashed line denotes the initiation threshold for the predictive forecast (post-2025). Higher resolution versions of all figures in the paper are included as Multimedia Appendix 1.

A differentiated treatment was applied to synonymous keyword terms. For the keyword cooccurrence network (Figure 1A, Multimedia Appendix 1) and the keyword frequency table (Table 1), original author-supplied terminology was preserved (eg, “chest X-ray,” “X-ray,” and “chest radiograph” were retained as separate entries) to track terminological evolution and disciplinary language preferences within the field. For the keyword burst analysis (Table 2), “chest X-ray” and “X-ray” were merged into a single “chest X-ray” entry to more accurately reflect the true burst intensity of the radiographic imaging theme and to avoid dilution of its thematic weight.

**Table 1.** Top 20 keywords by occurrence frequency and total link strength in AI-tuberculosis research. Total link strength represents the cumulative strength of cooccurrence relationships between keywords.

Rank	Keyword	Occurrences, n	Total link strength
1	Tuberculosis	348	764
2	Machine learning	248	474
3	Deep learning	233	631
4	Artificial intelligence	125	249
5	*Mycobacterium tuberculosis*	90	127
6	Chest X-ray	85	267
7	Convolutional neural network	77	225
8	Covid-19	70	224
9	Diagnosis	51	119
10	Pneumonia	46	167
11	Pulmonary tuberculosis	39	84
12	Classification	34	90
13	Feature extraction	34	151
14	Transfer learning	34	100
15	Support vector machine	33	81
16	Neural network	32	80
17	X-ray	30	161
18	Computed tomography	26	94
19	Biomarker	24	46
20	Chest radiograph	24	69

**Table 2.** Top 25 keywords with the strongest citation bursts in AI-Tuberculosis research^a^. (Source: CiteSpace [v.6.3.R3-6.4.R2, 64-bit Advanced], Web of Science Core Collection, search cutoff April 4, 2025).

Keyword	First year	Burst strength^b^	Burst begin^c^	Burst end^d^
Data mining	2004	6.40	2004	2017
Artificial neural networks	2006	4.87	2006	2018
Inhibitors	2009	4.86	2009	2014
*Mycobacterium tuberculosis*	2005	7.32	2010	2015
Identification	2006	4.13	2010	2014
Support vector machines	2012	6.55	2012	2017
Drug discovery	2012	5.45	2012	2019
Prediction	2012	3.36	2012	2017
Bayesian models	2013	6.44	2013	2017
Pharmacokinetics	2014	5.11	2014	2019
Multidrug resistant tuberculosis	2016	3.35	2016	2020
Support vector machine	2017	4.08	2017	2018
Mutations	2018	5.96	2018	2020
Drug resistance	2004	3.92	2018	2020
Database	2018	3.73	2018	2019
Hollow fiber model	2018	3.47	2018	2020
Combination	2019	4.13	2019	2020
Antibiotic resistance	2016	3.29	2019	2020
Protein	2020	4.89	2020	2021
Artificial intelligence	2020	3.42	2020	2021
Computed tomography	2021	4.04	2021	2023
Epidemiology	2004	3.88	2021	2022
Chest x-ray	2019	3.85	2022	2025
Radiography	2020	3.56	2022	2023
Pneumonia	2023	3.48	2023	2025

a Keywords with burst end=2025 are currently active research hotspots.

b Burst s trength: citation burst intensity score generated by CiteSpace.

c Burst begin: years of the burst period.

d Burst end: years of the burst period.

To project the future trajectory of the most active research topics, annual occurrence counts of the top 20 keywords from 2010 to 2025 were modeled using robust generalized additive models (GAM), and the fitted trends were extrapolated through 2027. Observed values are shown as points, fitted historical and projected trajectories as solid curves, and capped 95% CIs as shaded ribbons; a vertical dashed line indicates the forecast start.

### Ethical Considerations

Ethical approval was not required as this research involved exclusively secondary analysis of publicly available published literature from bibliographic databases, with no collection of primary data, no human subject involvement, and no access to personal or identifiable information. This determination aligns with standard international guidelines for bibliometric research that uses only published, publicly accessible academic literature.

## Results

### Publication and Citation Analysis

**Figure 2.**
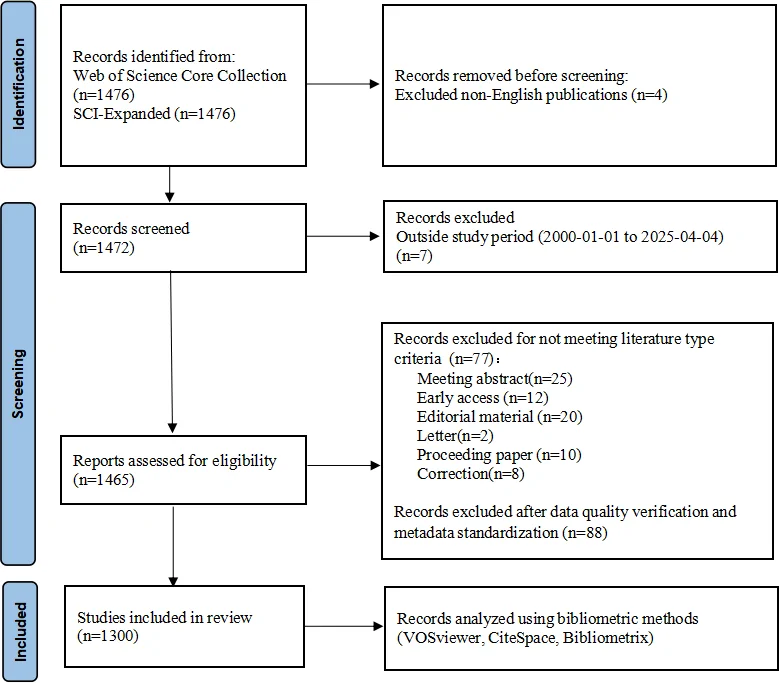
PRISMA (Preferred Reporting Items for Systematic Reviews and Meta-Analyses) 2020 flow diagram of literature identification and screening for AI-tuberculosis bibliometric analysis.

**Figure 3.**
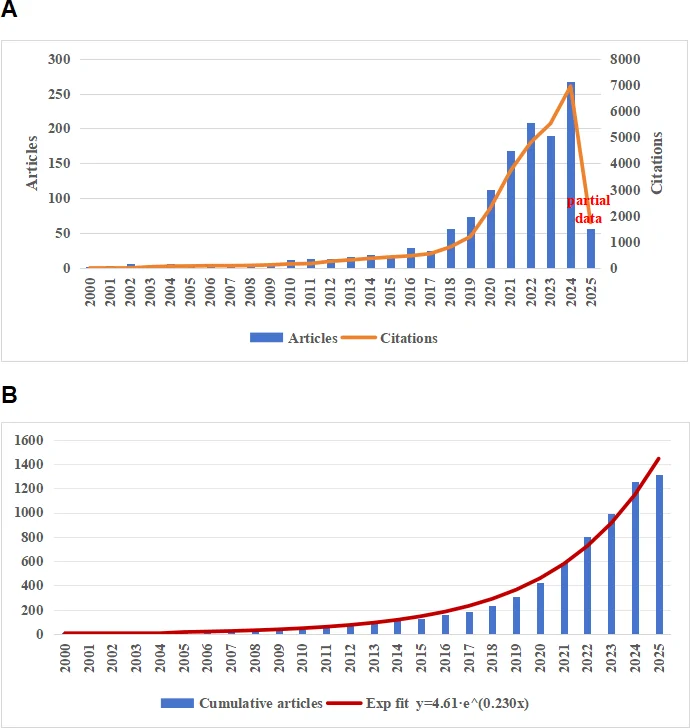
Annual and cumulative publication and citation trends in AI-tuberculosis research, 2000‐2025. (A) The trends in annual publication and citation counts in the field. The 2025 bar reflects partial-year data (January 1-April 4; n=56) and is excluded from growth rate calculations. (B) Exponential regression analysis of cumulative publication volume. Blue bars represent cumulative article counts; the red curve shows the exponential fit *y*=4.6083e^(0.2299·*x*), where *x* is the year index (*x*=0 corresponds to the year 2000), with parameters *a*=4.6083 and *b*=0.2299, fitted using Python 3 (SciPy curve_fit). The high coefficient of determination (*R*²=0.9865) confirms a strong model fit and validates the exponential growth trend in AI-tuberculosis research. Exp: exponential.

A total of 1300 articles from 167 countries and regions were retrieved (Figure 2). Annual publication and citation trends from 2000 to 2025 are shown in Figure 3A. Output remained low before 2010 (<10 publications and <150 citations per year), entered a sustained growth phase after 2018, and peaked in 2024 (267 publications and 6944 citations); the corresponding annual growth rates over 2018‐2024 were 29.7% for publications and 54.2% for citations. An exponential fit (*y*=4.6083e^(0.2299·*x*); *R*²=0.9865) closely matched the cumulative publication curve (Figure 3B), confirming an exponential growth trajectory.

### Country and Region Analysis

**Figure 4.**
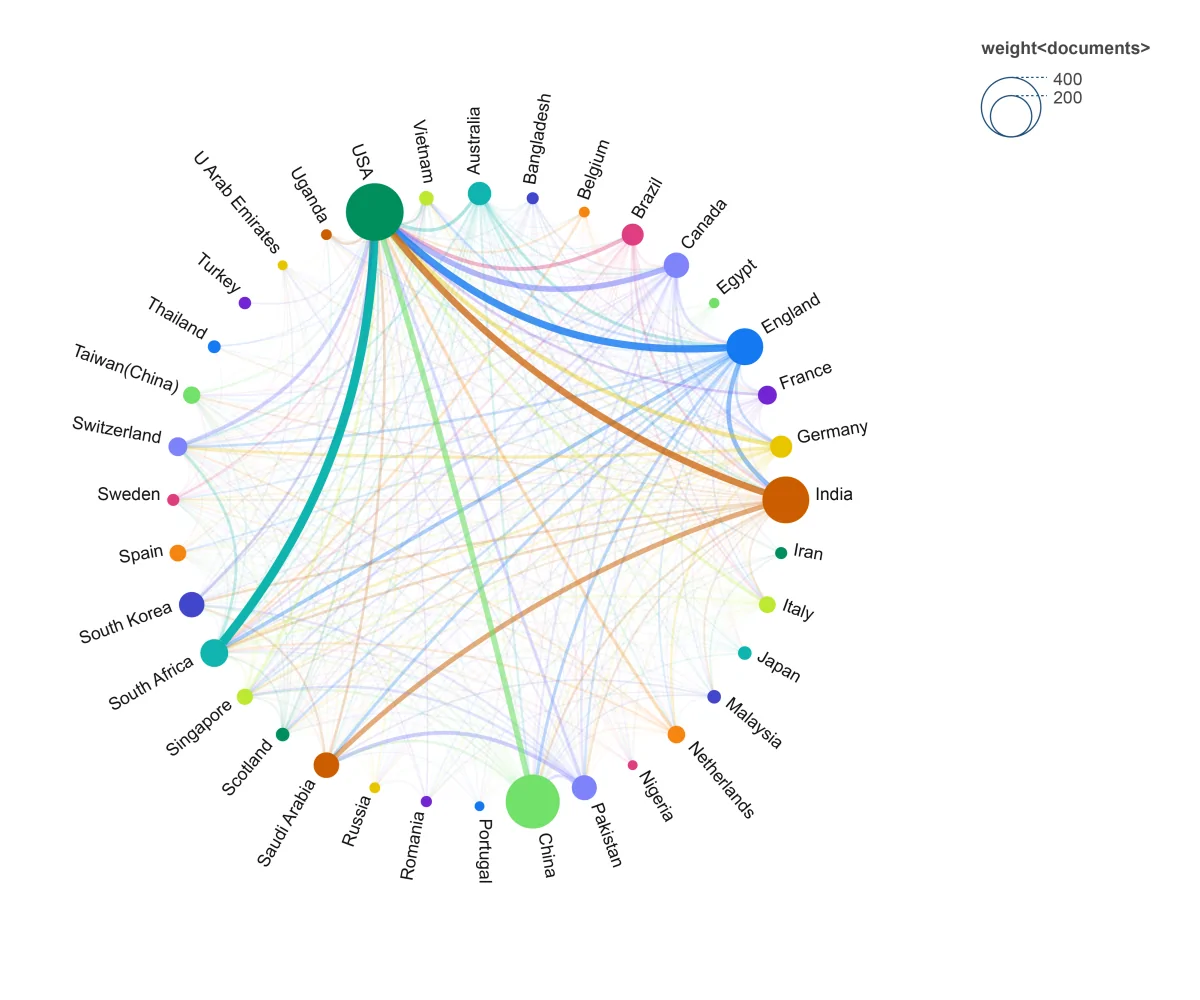
International collaboration network among countries and regions in AI-tuberculosis research, visualized using Scimago graphica.

The top 10 countries and regions by publication volume, citation count, and total link strength are summarized in Table 3. The top 3 by publication volume were the United States (n=346), China (n=304), and India (n=228). The United States led across all 3 bibliometric dimensions—publications, citations (n=12,143; 2.84-fold higher than second-ranked India), and total link strength (n=409)—consistent with its position as the field’s primary research hub. India ranked in the top 3 on every metric (third, second, and third), indicating a transition from scale expansion to quality enhancement. International collaboration patterns (Figure 4) confirm the central position of the United States and active participation by India, England, and South Africa, whereas China, despite ranking second in publication volume, shows comparatively limited international collaboration.

**Table 3.** Top 10 countries and regions by publication volume, citation count, and collaboration link strength in AI-tuberculosis research.

Rank	Country (by publications)	Publications, n	Country (by citations)	Citations, n	Country (by link strength)	Total link strength
1	United States	346	United States	12,143	United States	409
2	China	304	India	4280	England	233
3	India	228	China	4251	India	196
4	England	141	England	4090	South Africa	185
5	South Africa	80	Canada	3282	Canada	146
6	Saudi Arabia	68	South Africa	1728	China	141
7	Canada	67	Netherlands	1674	Germany	131
8	South Korea	67	Germany	1626	Pakistan	117
9	Pakistan	65	South Korea	1490	Australia	109
10	Australia	57	Australia	1314	Saudi Arabia	107

### Author Analysis

The top 10 authors by publication volume and citation count are listed in Tables 4 and 5. Sean Ekins led the publication ranking (n=27 papers), followed by Tawanda Gumbo (n=19) and Jotam G Pasipanodya (n=15), with 90% (9/10) of the top-ranked authors affiliated with US institutions. In the citation ranking, the WHO (n=438 citations) ranked first, reflecting the high cocitation frequency of its policy documents, followed by Sean Ekins (n=319) and Stefan Jaeger (n=269); Tawsifur Rahman (Qatar University; n=137 citations) and EJ Hwang (Seoul National University Hospital; n=129 citations) emerged as the principal Asian research nodes.

**Table 4.** Top 10 authors by publication volume in AI-tuberculosis research.

Rank	Author	Documents	Total link strength	Countries or regions	Institution
1	Ekins, Sean	27	51	United States	Collaborat Pharmaceutical Inc
2	Gumbo, Tawanda	19	40	United States	Praedicare Labs
3	Pasipanodya, Jotam G	15	34	United States	Vanderbilt University
4	Antani, Sameer	13	30	United States	National Institutes of Health
5	Freundlich, Joel S	13	31	United States	Rutgers University System
6	Jaeger, Stefan	11	32	United States	National Institutes of Health
7	Rajaraman, Sivaramakrishnan	10	26	United States	National Institutes of Health
8	Zhang, Yu-Dong	9	16	England	University of Leicester
9	Beamer, Gillian	8	20	United States	Aiforia Inc
10	Deshpande, Devyani	8	28	United States	Baylor University

**Table 5.** Top 10 authors by cocitation count in AI-tuberculosis research.

Rank	Author	Cocitations	Total link strength	Countries or regions	Institution
1	World Health Organization	438	2546	—^a^	—
2	Ekins, Sean	319	1608	United States	Collaborat Pharmaceutical Inc
3	Jaeger, Stefan	269	3687	United States	National Institutes of Health
4	Lakhani, Paras	145	2074	United States	Thomas Jefferson University
5	Rahman, Tawsifur	137	1862	Qatar	Qatar University
6	He, Kaiming	136	1583	United States	Massachusetts Institute of Technology
7	Hwang, Eui Jin	129	1839	South Korea	Seoul National University Hospital
8	Rajaraman, Sivaramakrishnan	128	2455	United States	National Institutes of Health
9	Breiman, Leo	124	744	United States	University of California Berkeley
10	Szegedy, Christian	119	1801	United States	xAI^b^

a Not applicable.

b xAI: experimental AI.

**Figure 5.**
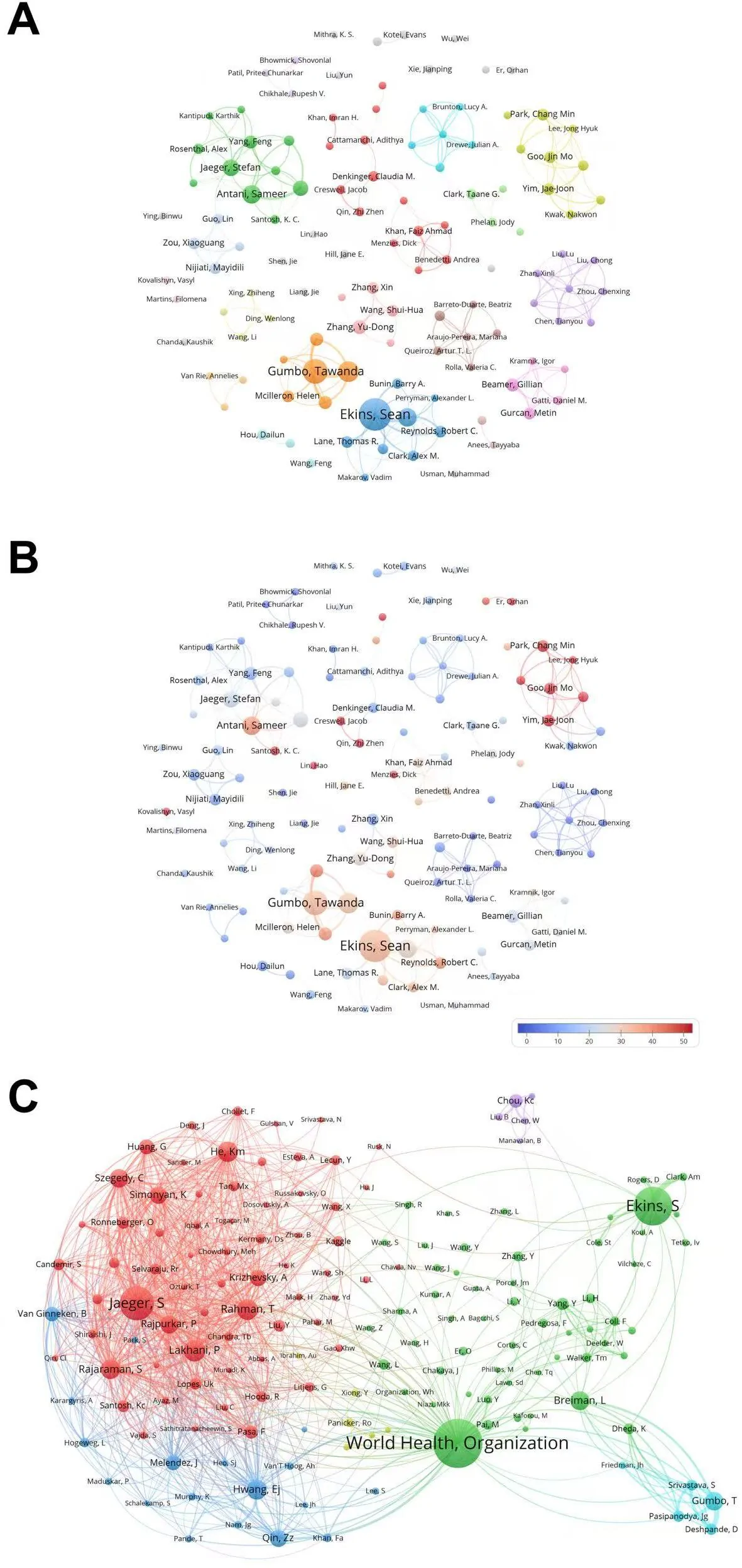
Author collaboration, citation intensity, and cocitation network in AI-tuberculosis research. (A) Author publication volume and collaboration relationships. The size of the nodes is positively correlated with publication volume, and the lines indicate collaborative relationships. (B) Visualization network of author collaboration intensity. Intensity overlap is shown based on Figure 5A, with red indicating higher intensity. (C) Analysis of cocitation relationships among authors. Clusters of different colors roughly represent the focus of various research directions in the field, with larger nodes indicating higher cocitation counts.

Three densely connected research communities are evident in the author collaboration network (Figure 5A), anchored by Sean Ekins, by Sameer Antani and Stefan Jaeger of the National Institutes of Health, and by Tawanda Gumbo; smaller but cohesive South Korean (Chang Min Park) and Chinese (Chong Liu) clusters are also present, while the remaining isolated nodes correspond to authors with minimal collaborative ties. Overlay analysis (Figure 5B) identifies the South Korean cluster led by Chang Min Park and Jin Mo Goo as currently the most active, marking it as an emerging research node. At the cocitation level (Figure 5C), 6 knowledge domains are resolved, of which 3 dominate: a diagnostic imaging cluster (Stefan Jaeger and Olaf Ronneberger) focused on radiographic and pathological slide recognition; a drug development cluster (Sean Ekins) covering AI-assisted treatment design, drug resistance prediction, and molecular screening; and a public-health cluster (Zhi Zhen Qin and Eui Jin Hwang) addressing epidemic forecasting, high-risk screening, and intervention optimization. The presence of the WHO as an institutional node in this network reflects the high cocitation frequency of its policy documents rather than authorship in the conventional sense.

WHO entries reflect the aggregated cocitation frequency of WHO policy documents, not authored research papers. They are retained to represent the field’s systematic reliance on international TB guidelines.

### Institutional Analysis

The top 10 institutions by publication volume and citation count are listed in Tables 6 and 7; institutions in the United States and England together accounted for more than 70% (15/20) of both metrics. The University of Cape Town (South Africa) led publication output (n=35 papers), followed by Harvard Medical School (n=24) and the London School of Hygiene and Tropical Medicine (n=22). Citation impact was dominated by US institutions, with Harvard Medical School (n=1693 citations), Stanford University (n=1433), and the Massachusetts Institute of Technology (n=1303) occupying the top 3 positions. A total of 3 institutions—Harvard Medical School, the University of Cape Town, and the London School of Hygiene and Tropical Medicine—achieved both substantial output and citation counts exceeding 1000, indicating a sustained balance of productivity and impact. A total of 2 Chinese institutions, Capital Medical University (n=19 papers) and the Collaborative Innovation Center of Chemistry for Energy Materials (n=15 papers), entered the top 10 by output but ranked lower in citation impact.

**Table 6.** Top 10 institutions by publication volume in AI-tuberculosis research.

Rank	Institution	Publications, n	Original country
1	University of Cape Town	35	South Africa
2	Harvard Medical School	24	United States
3	London School of Hygiene and Tropical Medicine	22	England
4	University College London	21	England
5	Capital Medical University	19	China
6	Johns Hopkins University	19	United States
7	National Institutes of Health	19	United States
8	University of Oxford	19	England
9	University of California, San Francisco	17	United States
10	Collaborative Innovation Center of Chemistry for Energy Materials	15	China

**Table 7.** Top 10 institutions by citation count in AI-tuberculosis research.

Rank	Institution	Citations, n	Original country
1	Harvard Medical School	1693	United States
2	Stanford University	1433	United States
3	Massachusetts Institute of Technology	1303	United States
4	Harvard University	1295	United States
5	University of Cape Town	1095	South Africa
6	London School of Hygiene and Tropical Medicine	1055	England
7	University of California, San Francisco	702	United States
8	Seoul National University	629	South Korea
9	National Institutes of Health	600	United States
10	University College London	572	England

**Figure 6.**
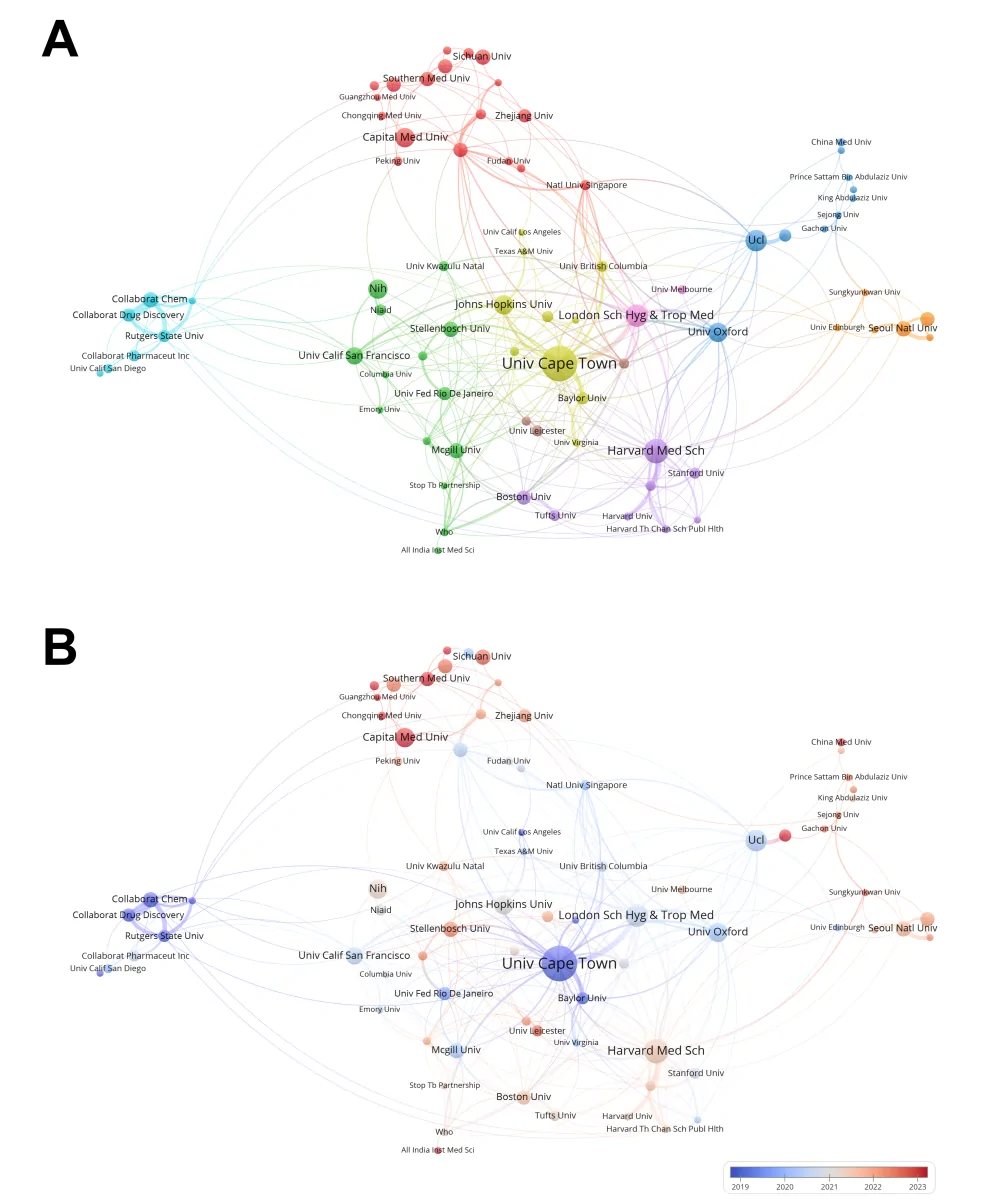
Institutional collaboration network and publication timeline in AI-tuberculosis research. (A) Collaboration relationships among research institutions in the field from 2000 to 2025. (B) Visualization analysis of institutional collaboration over time. Red indicates more recent research, while blue represents earlier publications. The legend spans 2019‐2023, reflecting the period of peak institutional publication activity.

Six regional clusters were observed in the institutional collaboration network (Figure 6A): a US cluster anchored by Harvard, Stanford, and Boston Universities; a Chinese cluster comprising Capital Medical, Zhejiang, Southern Medical, Sichuan, Peking, and Fudan Universities; a UK cluster led by University College London and the University of Oxford; a network centered on the University of Cape Town with extensive cross-cluster ties; and 2 international clusters bridging the US, Brazilian, South African, and South Korean institutions. Some labels are suppressed by VOSviewer’s overlap avoidance algorithm; the complete institution list is provided in Tables 6 and 7. Temporal overlay (Figure 6B) showed that early contributions originated from the University of Cape Town, the Collaborative Innovation Center of Chemistry for Energy Materials, and the London School of Hygiene and Tropical Medicine, whereas Chinese institutions (Capital Medical, Southern Medical, Sichuan, and Zhejiang Universities) have become increasingly active in recent years and represent emerging contributors to the field.

### Journal Analysis

The top 10 journals by publication volume and cocitation frequency are listed in Tables 8 and 9. *Scientific Reports* (IF=3.9; Journal Citation Reports [JCR] 2024 quartile 1) led publication output (n=49 articles), followed by *IEEE Access* (n=36; quartile 2), *Diagnostics* (n=34; quartile 1), and *PLoS One* (n=33; quartile 1); 90% of the top 10 publication volume journals were quartile 2 or above, and all top 10 cocited journals were quartile 1. *PLoS One* recorded the highest cocitation count (n=1442), followed by *Scientific Reports* (n=1195), with both exceeding 1000 cocitations.

**Table 8.** Top 10 journals by publication volume in AI-tuberculosis research, with IFs^a^ and JCR^b^ rankings.

Rank	Journal	Publications	IF (JCR 2024)	JCR quartile
1	*Scientific Reports*	49	3.9	Q1^c^
2	*IEEE Access*	36	3.6	Q2
3	*Diagnostics*	34	3.6	Q1
4	*PLoS One*	33	3.7	Q1
5	*Tuberculosis*	21	2.2	Q2
6	*Clinical Infectious Diseases*	15	8.2	Q1
7	*BMC Infectious Diseases*	14	3.4	Q2
8	*Frontiers in Microbiology*	14	4	Q2
9	*Applied Sciences*	13	2.5	Q2
10	*Computers in Biology and Medicine*	13	7	Q1

a IF: impact factor.

b JCR: Journal Citation Reports.

c Q: quartile.

**Table 9.** Top 10 journals by cocitation frequency in AI-tuberculosis research, with IFs^a^ and JCR^b^ rankings.

Rank	Cocited journal	Citations	IF (JCR 2024)	JCR quartile
1	*PLoS One*	1442	3.7	Q1^c^
2	*Scientific Reports*	1195	3.9	Q1
3	*Antimicrobial Agents and Chemotherapy*	836	4.1	Q1
4	*Proceedings of the IEEE Computer Society Conference on Computer Vision and Pattern Recognition*	777	—^d^	—
5	*Nucleic Acids Research*	747	16.7	Q1
6	*arXiv*	742	—	—
7	*Bioinformatics*	691	4.4	Q1
8	*Clinical Infectious Diseases*	635	8.2	Q1
9	*Radiology*	619	12.1	Q1
10	*International Journal of Tuberculosis and Lung Disease*	586	3.8	Q1

a IF: impact factor.

b JCR: Journal Citation Reports.

c Q quartile.

d Not applicable.

Notably, 2 non–peer-reviewed sources, *Proceedings CVPR IEEE Computer Society Conference on Computer Vision and Pattern Recognition* (n=777 citations) and *arXiv* (n=742 citations), also appeared in the cocitation list, reflecting the field’s reliance on rapid dissemination channels for emerging AI methodologies that typically appear in conference proceedings or preprint servers before formal journal publication.

**Figure 7.**
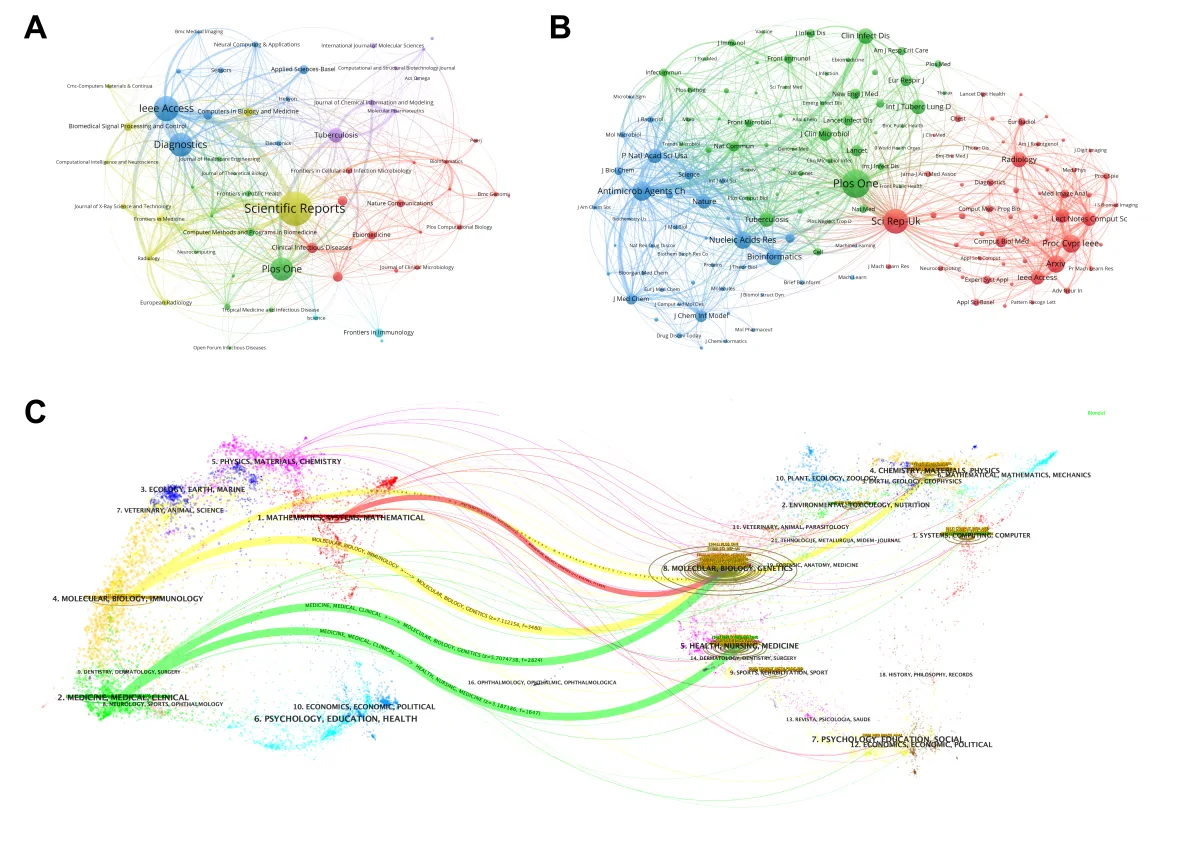
Journal copublication network, cocitation clustering, and dual-map overlay in AI-tuberculosis research. (A Visualization network of journal relationships in the field. The size of the nodes reflects publication volume, and the lines represent the degree of association, with thicker lines indicating stronger associations. (B) Cocitation analysis of journals. Different colors distinguish the focus areas of journal research, and the size of the nodes is positively correlated with cocitation counts. (C) Dual-mapping analysis of journals. Left: citing journals; right: cited journals, with the relationship between them indicated by the connecting lines.

A total of 6 thematic clusters emerged from the journal cooccurrence network (Figure 7A): computer-applied technologies (eg, *BMC Medical Imaging* and *Neural Computing and Applications*); medical imaging and radiology (eg, *Scientific Reports*, *Radiology*, and *European Radiology*); public health strategies (eg, *PLoS One* and *Frontiers in Public Health*); TB drug development (eg, *Tuberculosis* and *Journal of Chemical Information and Modeling*); bioinformatics (eg, *Nature Communications* and *Bioinformatics*); and immunology and cell biology (eg, *Frontiers in Immunology* and *iScience*). At the cocitation level (Figure 7B), these sources collapsed into 3 densely interconnected domains: infection and immunity, anchored by *PLoS One* (n=1442 citations) alongside *Journal of Infection* and *The Lancet Infectious Diseases*; medical imaging and computational biomedicine, represented by *Radiology*, *Medical Image Analysis*, and *Lecture Notes in Computer Science*; and microbiology, biochemistry, and pharmaceutical research, including *Journal of Medicinal Chemistry* and *Advanced Drug Delivery Reviews*. As shown by the dual-map overlay (Figure 7C), citing journals in biology, immunology, clinical medicine, and health care drew predominantly from cited journals in nursing, public health, molecular biology, and pharmacology—an established cross-disciplinary citation pattern.

### Keyword Analysis

The top 20 keywords by frequency and total link strength (TLS) are listed in Table 1. Overall, 3 terms ranked substantially above the rest—“tuberculosis” (n=348 occurrences; TLS=764), “machine learning” (n=248; TLS=474), and “deep learning” (n=233; TLS=631)—establishing them as the central topics of the field.

Cooccurrence mapping (Figure 1A) resolved 5 thematic clusters: intelligent medical image processing and deep learning models (eg, “deep learning,” “chest radiograph,” “convolutional neural network,” and “computer-aided diagnosis”); TB diagnosis, treatment, and clinical decision-making (eg, “treatment,” “diagnosis,” and “pulmonary tuberculosis”); TB drug development and molecular mechanisms (eg, “isoniazid,” “pharmacokinetics,”and “molecular docking”); disease detection technologies (eg, “biomedical imaging,” “feature extraction,” and “X-ray”); and a central hub anchored by “tuberculosis” linking the four peripheral domains. Among the 25 keywords with the highest burst intensity (Table 2), 2—“chest x-ray” (3.85) and “pneumonia” (3.48)—remained in active burst phase, indicating contemporary and likely near-term research hotspots.

To landscape the temporal evolution of AI-TB research priorities, we modeled the annual frequency trajectories of the top 20 keywords from 2010 to 2025 using robust GAM and projected research vitality through 2027 (Figure 1B). The macro-level pattern reveals a definitive transition from foundational biological terminology toward advanced computational frameworks. Traditional core terms—tuberculosis, *M. tuberculosis*, and pulmonary tuberculosis—exhibited sustained growth, with tuberculosis remaining the most dominant keyword and projected to maintain an upward trajectory. The prominence of “pulmonary tuberculosis” reflects the strong concentration of AI-TB research on pulmonary disease, particularly chest radiograph–based detection, whereas extrapulmonary tuberculosis remains comparatively underexplored. General computational concepts (machine learning, AI, and neural network) experienced steady growth through the 2010s, catalyzing the subsequent surge of deep learning and convolutional neural network (CNN), both exhibiting near-exponential growth from around 2018 with forecasts continuing to escalate through 2027.

From a clinical application perspective, chest x-ray (CXR) dominates imaging-based AI literature with a sharply accelerating trend, while computed tomography (CT) shows consistent increments. Post-2020, COVID-19 entered abruptly before stabilizing, reflecting the field’s rapid adaptation to coinfection differential diagnosis. Notably, transfer learning demonstrates strong upward momentum, highlighting a methodological consensus for addressing limited annotated TB imaging datasets. Conversely, support vector machine shows signs of plateauing, reinforcing the definitive shift toward end-to-end deep learning pipelines.

### Highly Cited Literature Analysis

The 15 most-cited papers in AI-TB research are summarized in Table 10. Stokes et al (Cell, 2020) [20] ranked first with 1110 citations, proposing a deep learning approach to antibiotic discovery that addresses efficiency bottlenecks in traditional drug development. A total of 2 further papers exceeded 700 citations: Lakhani and Sundaram (Radiology, 2017) [21], who pioneered convolutional neural network–based classification of pulmonary tuberculosis on chest radiographs (1015 citations), and Rajpurkar et al (*PLoS Medicine*, 2018) [22], a retrospective comparison of the CheXNeXt algorithm with practicing radiologists (701 citations).

**Table 10.** Top 15 most-cited publications in AI-tuberculosis research with citation counts.

Rank	Author	Article title	Journal title	Citation counts, n	Year	Document type
1	Stokes et al [20]	A deep learning approach to antibiotic discovery	*Cell*	1110	2020	Article
2	Lakhani and Sundaram [21]	Deep learning at chest radiography: automated classification of pulmonary tuberculosis by using convolutional neural networks	*Radiology*	1015	2017	Article
3	Rajpurkar et al [22]	Deep learning for chest radiograph diagnosis: a retrospective comparison of the CheXNeXt algorithm to practicing radiologists	*PLoS Medicine*	701	2018	Article
4	Alcock et al [23]	CARD 2023: expanded curation, support for machine learning, and resistome prediction at the Comprehensive Antibiotic Resistance Database	*Nucleic Acids Research*	614	2023	Article
5	Bendtsen et al [24]	Non-classical protein secretion in bacteria	*BMC Microbiology*	559	2005	Article
6	Warnat-Herresthal et al [25]	Swarm learning for decentralized and confidential clinical machine learning	*Nature*	455	2021	Article
7	Dhanda et al [26]	Designing of interferon-gamma inducing MHC class-II binders	*Biology Direct*	436	2013	Article
8	Schwalbe and Wahl [27]	Artificial intelligence and the future of global health	*Lancet*	320	2020	Review
9	Hwang et al [28]	Development and validation of a deep learning-based automated detection algorithm for major thoracic diseases on chest radiographs	*JAMA Network Open*	274	2019	Article
10	Peiffer-Smadja et al [29]	Machine learning for clinical decision support in infectious diseases: a narrative review of current applications	*Clinical Microbiology and Infection*	261	2020	Review
11	Suet al [30]	Genome-based prediction of bacterial antibiotic resistance	*Journal of Clinical Microbiology*	227	2019	Review
12	Agranoff et al [31]	Identification of diagnostic markers for tuberculosis by proteomic fingerprinting of serum	*Lancet*	227	2006	Article
13	Rahman et al [32]	Reliable tuberculosis detection using chest X-ray with deep learning, segmentation and visualization	*IEEE Access*	223	2020	Article
14	Korotcov et al [33]	Comparison of deep learning with multiple machine learning methods and metrics using diverse drug discovery data sets	*Molecular Pharmaceutics*	222	2017	Article
15	Oruç et al [34]	1,3,4-thiadiazole derivatives: synthesis, structure elucidation, and structure-antituberculosis activity relationship investigation	*Journal of Medicinal Chemistry*	222	2004	Article

**Figure 8.**
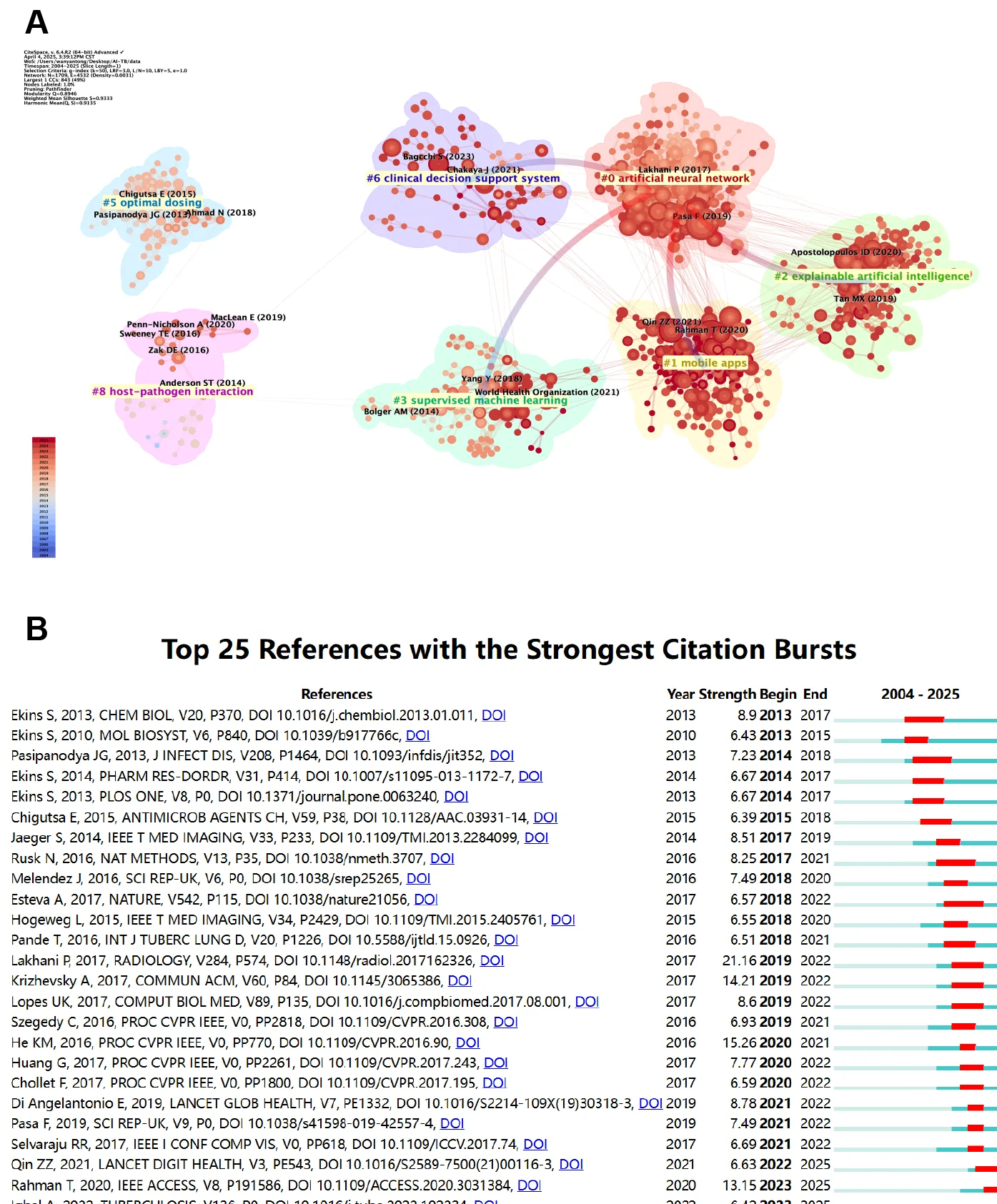
Cocitation clustering and burst analysis of highly cited literature in AI-tuberculosis research. (A) Network of the most cited literature in the field combined with time. Red indicates more recent research, with nodes representing the first authors of highly cited papers. Clusters #4 and #7 were automatically merged into adjacent clusters by the algorithm due to insufficient node frequency. (B) Intensity of the outbreak and duration of highly cited literature [21,32,36-58].

Reference cocitation analysis (Figure 8A) resolved 7 thematic clusters (#0–#6, plus #8), among which #0 artificial neural networks was the largest. The research focus has shifted in recent years toward #0 artificial neural networks, #1 mobile apps, and #2 explainable AI, marking these as near-term research priorities; emerging large language model (LLM) applications represent a particularly promising frontier, with a recent clinical evaluation showing that LLMs provide accurate TB-related health consultation and patient education across diagnosis, treatment, and prognosis domains [35]. Earlier research, by contrast, was concentrated in clusters #5 (optimal dosing) and #8 (host-pathogen interaction), most of which date to 2019 or earlier and constitute the foundational layer of the field.

Citation burst analysis is shown in Figure 8B [21,32,36-58]. Lakhani and Sundaram [21] combined the highest citation count (1015) with the strongest burst intensity (21.16), reflecting a sustained leading role in the field’s development. Three papers remained in active burst phase at the time of analysis: Qin et al (*Lancet Digital Health*; burst intensity 6.63) [36], Rahman et al (*IEEE Access*, 2020; 13.15) [32], and Iqbal et al (*Tuberculosis*, 2022; 6.42) [37]. All 3 address deep learning–based TB detection on chest radiographs, indicating that radiographic AI screening remains the dominant near-term research frontier.

## Discussion

### Principal Findings

Bibliometric analysis of 1300 AI-tuberculosis publications (2000‐2025) reveals a field marked by exponential growth yet structural imbalance: research investment concentrates in imaging diagnostics and drug discovery dominated by high-income countries, while clinical deployment validation and public health applications in high-burden settings remain critically underdeveloped. This research-demand inversion constitutes the central tension examined across the following sections.

### Research Development Trajectory and Global Landscape Analysis

This study used bibliometric methods to systematically analyze the application of AI in tuberculosis management from 2000 to 2025. The analysis results demonstrate that AI-TB research has experienced distinct developmental phase differentiation, with 2018 serving as a critical turning point, after which the field entered a rapid growth period with an annual publication growth rate of 29.7% and a citation growth rate of 54.2% (see the Publication and Citation Analysis subsection above), exhibiting strong developmental momentum (Figure 3A). This growth trajectory is closely related to the synergistic effects of multiple factors, particularly the WHO’s continued emphasis on technology-driven precision medicine needs in TB prevention and control policies [1], which has created a favorable environment for the flourishing development of interdisciplinary research.

### Geographic Concentration of Global Research Leadership

As demonstrated in Table 3, the United States occupies the leading position across all 3 dimensions. This dominance is not coincidental but reflects systematic advantages in resource allocation. Analysis by Wahls et al [59] of National Institutes of Health funding distribution reveals significant Matthew effects in both geographic and institutional dimensions, with researchers in advantaged regions showing markedly higher funding acquisition rates and application success rates compared to other regions.

Personnel distribution data further confirms this resource concentration effect: 90% of the top 10 prolific authors in this field are affiliated with US institutions (Tables 4 and 5). Leading research institutions such as Harvard Medical School, Stanford University, and the Massachusetts Institute of Technology not only play a leading role in technological innovation but also possess significant influence in discipline development direction and research standard formulation (Tables 6 and 7). This phenomenon reflects the typical manifestation of the “Matthew effect” described by Merton in academic resource allocation [60].

However, when we contrast this academic prosperity with actual disease burden, a thought-provoking contradiction emerges. The research activity level in the United States forms a stark contrast with its relatively low disease burden: the US tuberculosis incidence rate is only 2.9 per 100,000 population, far below the global average. In comparison, India, despite producing 228 papers, has an incidence rate as high as 195 per 100,000 population, approximately 67 times that of the United States; South Africa produced only 80 papers but has an incidence rate of 468 per 100,000 population, which is approximately 161 times that of the United States and 2.4 times that of India [1].

This phenomenon embodies the well-known “research-need inversion” pattern in global health research, where regions with the strongest research capabilities are often not those with the heaviest disease burden. This observation is highly consistent with the “10/90 gap” phenomenon in global health inequality research—although the precision of this ratio estimate is debatable, the overall trend it reflects remains significant: only about 10% of global biomedical research budgets are allocated to addressing health problems that account for 90% of the global disease burden [61]. A recent scoping review of AI applications in TB control corroborates this pattern, finding that the majority of current AI-TB work remains at early validation stages with limited translation to high-burden settings [62].

### Systematic Challenges and Exploration of Diversified Collaboration Models

This geographic mismatch between “high-technology-low-burden” and “low-technology-high-burden” brings systemic risks. Countries with the heaviest tuberculosis burden typically lack the technological infrastructure to develop AI solutions, while countries with advanced AI capabilities have relatively limited understanding of the complex clinical realities of tuberculosis [63]. This disconnect between development and deployment regions, combined with the complexity of cross-national data acquisition, may constrain the optimal utility of AI technology in actual disease control [64].

However, these challenges have also catalyzed active exploration of new global collaboration models. The unique strategic value demonstrated by England merits particular attention; despite its TLS of 233 being lower than the United States’ 409, the United Kingdom ranks 4th in citation impact (4090 citations; Table 3) and maintains close connections with numerous high-burden countries in international collaboration networks (Figure 4). The success of this model is primarily attributed to institutions such as the London School of Hygiene and Tropical Medicine that have long been committed to establishing deep collaborative relationships with high-burden countries in Africa and Asia [65] (Tables 6 and 7).

China presents another developmental trajectory, characterized by “internally strong, externally weak” features. China ranks second globally with 304 papers, but its international collaboration intensity (141) and academic impact (4251 citations) still show significant gaps compared to the United States (Table 3). Its collaboration network is primarily concentrated among domestic institutions. While this relatively independent development path may limit the integration of diverse international perspectives, it also demonstrates the possibility of constructing a relatively autonomous research ecosystem [64,65].

Meanwhile, India, as a high-burden country, has achieved a breakthrough by ranking second globally in citation impact (Table 3), demonstrating the positive efforts of disease-burden regions to enhance academic discourse power through improving research quality. The University of Cape Town in South Africa achieved 1095 citations with 35 papers (Tables 6 and 7), reflecting the strategic choice of high-burden regions to build regional centers of research excellence by concentrating limited resources.

### Resource Allocation Mechanisms and Academic Publishing Ecosystem Analysis

The underlying mechanisms behind these diverse development models merit in-depth analysis. The 2024 Global Innovation Index by the World Intellectual Property Organization shows that the United States is 1 of 9 countries globally with R and D (research and development) investment exceeding 3% of GDP (gross domestic product), a level of investment far surpassing most developing countries [66]. Fundamental differences in funding capacity directly translate into differences in research quality and impact: United States published papers average 35.1 citations, while papers from India and China average 18.8 and 14.0 citations respectively (Table 3).

More critically, funding structures themselves shape research priorities. High-income countries rely on sustained government investment and private innovation funding, enabling support for basic algorithmic research that requires long-term investment. In contrast, low- and middle-income countries mainly depend on international aid and short-term project funding, directing their research priorities toward more immediate clinical application needs [67,68].

The academic publishing system further reinforces this resource allocation imbalance. Journal distribution patterns provide important publishing ecological evidence for understanding research-need mismatch (Figure 7A B). Technology-oriented journals such as *Scientific Reports* and *IEEE Access* dominate in publication numbers, primarily focusing on algorithmic model development and medical imaging diagnostic technology optimization, while journals truly focused on clinical validation and practical application challenges publish relatively fewer papers (Tables 8 and 9).

High-impact comprehensive journals (such as *Cell and Radiology*; Table 10) show clear preferences for technological breakthroughs, with limited attention to research addressing practical deployment issues in resource-constrained environments. This journal orientation forms a complete incentive chain from funding input to academic evaluation, further reinforcing the value orientation that prioritizes algorithmic innovation over clinical translation [69].

Notably, the WHO 2024 report indicates that global tuberculosis research and development (R and D) funding in 2021 totaled only US $1 billion, representing a huge gap from the US $5 billion target for 2027 [1]. This funding shortage not only reflects the serious inadequacy of current investment but also provides an opportunity window for redesigning more balanced funding allocation mechanisms.

### Research Focus Evolution and Technology-Society Integration Trends

Through systematic analysis of the research focus in highly cited literature, we find that the field exhibits pronounced technological concentration, with algorithmic innovation as the dominant research paradigm (Table 10). This concentration also exposes structural problems that warrant closer examination.

This concentration reveals distinct translational gaps across application domains. In diagnostics, 4 of 15 (26.7%) highly cited papers develop imaging algorithms, yet none of these primarily validate deployment effectiveness in primary care settings, exposing insufficient generalizability testing across diverse equipment and operator skill levels. Therapeutic applications face greater translational challenges: 2 papers (2/15, 13.3%) focus on drug-resistance prediction and 3/15 (20%) on drug discovery, but virtually no studies evaluate how these predictive models integrate into existing clinical decision workflows. Clinical validation studies confirm that even well-performing CAD algorithms require context-specific threshold calibration before deployment [70], while AI-driven drug-resistance prediction tools remain at the algorithmic development stage with limited clinical workflow integration [71]. Public health applications show the weakest translation of all: none of the 15 most-cited papers primarily addresses epidemic forecasting or resource allocation, and most work in this direction remains at proof-of-concept stages. Beyond these 3 domains, the remaining literature (6 of 15 papers) centers on foundational biology, decentralized-learning methodology, immunoinformatics, and narrative reviews—influential work that does not target a specific TB clinical application. This pattern reflects a fundamental paradigm issue: research simplifies tuberculosis into a technical problem while ignoring systemic implementation barriers including infrastructure limitations, data governance challenges, and the absence of socioeconomic factor integration (Table 10). This imbalance, where technical-validation-stage work far outweighs papers reporting clinical application, indicates that even when technology is relatively mature, academic attention to practical translation remains insufficient.

More concerning is the systematic absence of socioeconomic dimensions [72]. From the highly cited literature analysis in Table 10, it can be seen that literature explicitly incorporating key factors such as patient compliance, health care resource accessibility, and socioeconomic status into core research frameworks is extremely scarce. This low integration level not only explains the disconnect between technological innovation and actual needs but also explains why many AI solutions that perform excellently technically encounter difficulties in real-world deployment [73,74].

### Professional Differentiation and Development Imbalance From Network Analysis Perspective

Through in-depth analysis of keyword cooccurrence networks and author collaboration networks, we can more clearly understand the internal organizational logic of technology orientation. The dominance of algorithmic terminology in keyword analysis (Table 1) not only confirms the centrality of algorithmic innovation but, more importantly, reflects the research community’s collective focus on performance optimization.

The high-frequency cooccurrence of “chest X-ray” (85 times) and “convolutional neural network” (77 times; Table 1) further reveals a key technological development pattern: research priorities are highly focused on specific technology-application combinations, namely the application of deep learning algorithms in medical image analysis (Figure 1A). This focus both reflects the path-dependent characteristics of technological development [75,76] and the clustering effects of research resource concentration toward mature technological fields [77]. The post-2018 surge of deep learning and convolutional neural network alongside the plateau of support vector machine (Figure 1B) marks a shift from feature-engineering to end-to-end models, while the parallel rise of transfer learning reflects a community-level response to the chronic scarcity of annotated TB imaging datasets.

Author collaboration network analysis (Figure 5A) shows clear professional cluster differentiation, forming 3 relatively independent but differently mature research ecosystems. The medical imaging analysis ecosystem centered on the Antani and Jaeger teams has developed into a mature research network focused on continuous optimization of x-ray and CT image recognition technologies. Pioneering research by Lakhani et al [21] (burst strength 21.16; Figure 8B) and the segmentation visualization enhancement methods of Rahman et al [32] (burst strength 13.15; Figure 8B) have laid solid algorithmic foundations for this field.

The drug development ecosystem led by the Sean Ekins team is currently in a rapid expansion phase, forming an active innovation network around AI-assisted drug design and molecular screening algorithms (Figure 5B). Research on deep learning antibiotic discovery by Stokes et al [20] and continued contributions from the Sean Ekins team (319 citations; Tables 4 and 5) [78,79] fully demonstrate the breakthrough potential of AI technology in drug development.

In contrast, the public health application ecosystem with Qin ZZ and other researchers as nodes (Figure 5C), while relatively small in scale, represents emerging application directions for AI technology in epidemic prediction and high-risk population screening. Author cocitation network analysis (Figure 5C) identifies 6 research directions, but medical imaging analysis, drug development, and public health applications constitute the 3 major clusters, occupying the core position of research activities in this field.

### COVID-19 Pandemic Paradigm Insights and Future Development Opportunities

The COVID-19 pandemic offered an instructive paradigm for AI-TB research. In our keyword analysis, “COVID-19” appeared 70 times (Table 1), pointing to possible trajectories of technological development. The crisis-driven innovation model that emerged during the pandemic shows how research priorities can be rapidly restructured and interdisciplinary integration accelerated when technological innovation directly addresses urgent social needs. The swarm-learning framework developed by Warnat-Herresthal et al [25] (Table 10) is a representative case: rather than pursuing predictive accuracy alone, it incorporated real-world constraints—data privacy, resource disparities, and implementation feasibility—into its core design. This paradigm suggests that AI-TB research could shift from a technology-driven to a need-driven orientation under appropriate conditions, and that when research prioritizes practical problem-solving, both the efficiency and applicability of innovation may improve. Current trends suggest that public health applications may develop more rapidly in the coming years, particularly in socioeconomic-factor integration and deployment validation. This momentum stems both from improving technological maturity and from a shifting recognition of application value within the research community. Notably, a few studies have begun to integrate socioeconomic variables; for example, Qin et al [36] validated the effectiveness of AI chest-radiograph triage systems in high-burden regions. Although still a minority, the attention these studies have received signals a shift from purely technical tools toward decision-support systems that account for social context.

As the technological dividends in medical imaging analysis and drug development fields gradually approach diminishing marginal returns, research attention and resource allocation are likely to naturally shift toward public health applications, a relatively underdeveloped but highly promising field. This transformation not only promises to alleviate current technology-need mismatch problems but may also establish new development paradigms for global health technology innovation.

### Pathways to Clinical Translation

Resolving the research–demand inversion documented above requires coordinated action beyond technical refinement. On the policy side, national TB programs in high-burden countries could accelerate integration of validated chest-radiograph CAD software into screening guidelines, consistent with the 2021 WHO recommendation supporting CAD as an alternative to human reading for individuals aged 15 years or older [3], and funding agencies could ring-fence a defined share of AI-for-TB grants for prospective clinical evaluation rather than further algorithmic refinement. On the collaboration side, the weakly linked AI-engineering, clinical-medicine, and public-health clusters identified in our author cocitation analysis (Figure 5C) point to a need for deliberate team-science models, joint training programs producing hybrid specialists in machine learning and TB epidemiology, and standing advisory panels coled by computer scientists and frontline clinicians from high-burden settings. Complementary measures, including federated data infrastructure built on COVID-19–era governance templates [25] and clinician-driven postdeployment surveillance, would further support a transition from algorithm-development to deployment-oriented research. Building on these gaps, we identify 3 near-term research priorities: (1) randomized controlled trials of large language model–based education and adherence support for patients with TB in high-burden settings; (2) multisite validation of chest-radiograph CAD tools across heterogeneous hardware and operator skill levels; and (3) AI models integrating socioeconomic determinants into TB risk stratification and resource allocation.

### Limitations

This study has 3 primary limitations. First, exclusive reliance on the WOSCC and English-language publications likely underrepresents work from high-burden regions and may exaggerate the apparent US or European dominance in country-level analyses; future studies should extend the search to PubMed, Scopus, and regional indexes (eg, Latin American and Caribbean Health Sciences Literature and China National Knowledge Infrastructure). Second, citation-window bias inevitably undervalues recent (2022‐2025) work, including 2022 CAD validations [70] and emerging LLM applications [35]; presenting annual publications and citations side-by-side (Figure 3A) partially mitigates this, and field-weighted citation impact or Altmetric scores could further correct for it. Third, bibliometric proxies measure academic visibility rather than clinical value, so the “research–demand inversion” pattern (see Research Focus Evolution and Technology-Society Integration Trends above) should be interpreted structurally rather than evaluatively; future work pairing this mapping with clinical-expert Delphi surveys and implementation-science case studies would strengthen the inference.

### Conclusions

This study reveals a paradox in AI-TB research: a systematic inverse relationship between the geographic centers of technological innovation and the distribution of disease burden, forming a global research-demand inversion. This misalignment reflects an overemphasis on algorithmic optimization within academic incentive structures and limitations in how technologically advanced regions address complex social health problems. However, diversified development models are reshaping this landscape: bridging collaborations, independent research ecosystem development, and targeted breakthrough strategies in high-burden regions collectively offer pathways to break the dominant paradigm. The COVID-19 pandemic’s crisis-driven innovation model demonstrates that research focus can shift rapidly when technological innovation directly addresses urgent social needs. As traditional technological domains mature, public health applications’ potential creates opportunities for transitioning from a technology-driven to a need-driven orientation. AI-TB research stands at a critical juncture in global health technology innovation. Its successful transformation could advance control of this disease and inform more equitable, sustainable models of global health innovation.

## Supplementary material

10.2196/93885 Multimedia Appendix 1 Higher-resolution versions of Figures 1-8.

## Abbreviations

CAD: computer-aided detection
CNN: convolutional neural network
CT: computed tomography
CXR: Chest x-ray
GAM: generalized additive model
GDP: gross domestic product
IF: impact factor
JCR: Journal Citation Reports
LLM: large language model
LLR: log-likelihood ratio
PRISMA: Preferred Reporting Items for Systematic Reviews and Meta-Analyses
TB: tuberculosis
TLS: total link strength
WHO: World Health Organization
WOSCC: Web of Science Core Collection
